# *LGG* promotes activation of intestinal ILC3 through TLR2 receptor and inhibits *salmonella typhimurium* infection in mice

**DOI:** 10.1080/21505594.2024.2384553

**Published:** 2024-07-30

**Authors:** Junhong Wang, Ming Gao, Jiarui Wang, Yan Zeng, Chunfeng Wang, Xin Cao

**Affiliations:** a College of Veterinary Medicine, Jilin Agricultural University, Changchun, China; b Jilin Provincial Engineering Research Center of Animal Probiotics, Jilin Agricultural University, Changchun, China; c Jilin Provincial Key Laboratory of Animal Microecology and Healthy Breeding, Jilin Agricultural University, Changchun, China; d Engineering Research Center of Microecological Vaccines (Drugs) for Major Animal Diseases, Ministry of Education, Jilin Agricultural University, Changchun, China

**Keywords:** *Salmonella typhimurium*, lacticaseibacillus rhamnosus GG, type 3 innate lymphoid cells, intestine, infection

## Abstract

*Salmonella* is a foodborne pathogen that causes disruption of intestinal mucosal immunity, leading to acute gastroenteritis in the host. In this study, we found that *Salmonella Typhimurium* (*STM*) infection of the intestinal tract of mice led to a significant increase in the proportion of *Lacticaseibacillus*, while the secretion of IL-22 from type 3 innate lymphoid cells (ILC3) increased significantly. Feeding *Lacticaseibacillus rhamnosus GG* (*LGG*) effectively alleviated the infection of *STM* in the mouse intestines. TLR2^−/−^ mice experiments found that TLR2-expressing dendritic cells (DCs) are crucial for *LGG*‘s activation of ILC3. Subsequent in vitro experiments showed that heat-killed *LGG* (HK*-LGG*) could promote DCs to secrete IL-23, which in turn further promotes the activation of ILC3 and the secretion of IL-22. Finally, organoid experiments further verified that IL-22 secreted by ILC3 can enhance the intestinal mucosal immune barrier and inhibit *STM* infection. This study demonstrates that oral administration of *LGG* is a potential method for inhibiting *STM* infection.

## Introduction

The symbiotic relationship between intestinal probiotics and the host contributes to the maturation of intestinal function and the development of the immune system. Among probiotics, *Lacticaseibacillus rhamnosus GG* (*LGG*) is one of the most extensively studied. *LGG* is a Gram-positive beneficial bacterium present in the human gut, exhibiting strong adhesion to intestinal cells [1]. *LGG* can enhance the proliferation and differentiation of mouse intestinal epithelial cells, thereby strengthening the intestinal immune barrier [2]. Studies have shown that *LGG* can promote intestinal B cell development in piglets by the secreted p40 protein [3]. Studies have shown that indole substances in *LGG* metabolites can promote AhR receptor response to enhance intestinal immunity [4]. Moreover, *LGG* can inhibit pathogen infection by reducing intestinal pH, competing with pathogens for nutrients, and producing antimicrobial substances [5,6]. *LGG* can also activate macrophage activation by releasing lipoteichoic acid, promote the migration of mesenchymal stem cells, and ultimately protect the intestinal epithelium from radiation damage [7]. These studies found that *LGG* itself, secreted proteins and metabolites may have an important role in promoting intestinal immune development and disease prevention.

*Salmonella Typhimurium* (*STM*) is one of the primary pathogens responsible for acute gastroenteritis in hosts, capable of infecting various poultry and mammals, as well as humans [8]. *STM* infections can afflict the ileum and colon, and even the entire gastrointestinal tract of humans, posing serious health risks. In the early stages of *STM* infection, it can induce intestinal inflammation through direct infection of the intestinal barrier and destruction of intestinal epithelial cells [9]. Some studies have reported that *LGG* can inhibit *Salmonella* infection by regulating macrophages [10]. In this study, we further explored the pathways by which *LGG* activates intestinal immunity to inhibit *STM* infection.

Innate Lymphoid Cells (ILCs) are a newly discovered class of lymphocytes involved in innate immunity, playing a crucial role in protecting tissue health and combating infections. Among them, ILC3 plays a key role in maintaining homoeostasis within the intestine. They respond to various dietary components and microbes both inside and outside the body, can sense changes in the gut microbiome, thereby regulating intestinal immune responses and maintaining the stability of the gut microbiota [11,12]. ILC3s can secrete cytokines such as IL-22 and IL-17 to regulate intestinal mucosal immunity, not only interacting with intestinal stem cells to regulate their differentiation and function but also playing a crucial role in tissue repair processes [13–15]. A study elucidated the mechanism behind ILC3-driven intestinal tissue repair, revealing that ILC3 can stimulate epithelial cell proliferation and tissue regeneration by activating the Yap1 signalling pathway in intestinal crypt cells [16]. ILC3s can regulate epithelial cells through IL-22 signalling, including the expression of tight junction proteins, the expression of Major Histocompatibility Complex II (MHC-II), and the production of antimicrobial peptides [17,18]. ILC3s are also capable of inhibiting intestinal epithelial cell death and maintaining barrier integrity under the regulation of chemokine CCL3 and T cells [19]. Recent research findings have clarified that innate immune cells, such as dendritic cells (DCs) and macrophages, can regulate ILC3 through the production of IL-1β and IL-23 [20]. Most importantly, recent studies have found that ILC3 can inhibit Salmonella infection [21]. In summary, we mainly studied whether feeding *LGG* could regulate the gut microbiota and enhance intestinal mucosal immunity by activating ILC3, ultimately inhibiting *STM* infection.

Toll-like receptors (TLRs) are a class of pattern recognition receptors (PRRs) in the innate immune system responsible for the early detection of invading pathogens [22]. Among them, TLR2 is expressed on a variety of cell types, including immune cells, endothelial cells, and epithelial cells [23]. The expression of TLR2 on DCs can regulate T cells’ response to Th2, induce the proliferation of CD4^+^CD25^+^Foxp3^+^ T cells, and promote the production of IL-10 and TGF-β by T cells [24]. TLR2 has been shown to play a protective role during infections by triggering a strong pro-inflammatory response [25]. Studies have demonstrated that in a mouse model of Mycobacterium tuberculosis infection, activation of TLR2 on CD4^+^ T cells leads to an increase in the protective IFN-γ secretion by T cells [26]. Moreover, administering TLR2 agonists can enhance the phagocytic action and bactericidal activity of neutrophils, thereby protecting mice from infection with *Methicillin-resistant Staphylococcus aureus* (*MRSA*) [27]. In summary, TLR2 is closely related to the recognition of the gut microbiota, and whether DC expressing TLR2 plays a role in inhibiting STM infection deserves further exploration.

In this study, we found that feeding mice with *LGG* significantly inhibited infection by *STM*. Subsequent experiments with TLR2^−/−^ mice and in vitro cell studies revealed that heat-killed *LGG* (HK-*LGG*) activates ILC3 through DCs. It is conjectured that IL-22 secreted by ILC3 plays a crucial role in maintaining intestinal antibacterial functions and development, which can enhance the intestinal mucosal immune barrier and promote organoid development.

## Materials and methods

### Animals, and ethical statement

Wildtype mice (C57BL/6) used in this experiment were purchased from HFK Bioscience Co., Beijing, China. TLR2^−/−^ mice (C57BL/6) purchased from Cyagen Biotechnology Co., Suzhou, China. For the duration of the experiments, all animals were accommodated in SPFgrade animal houses within a specific pathogen-free facility. The housing conditions included a 12-hour light/dark cycle and maintained appropriate ambient temperature and humidity levels. The entire animal experiment complied with the requirements of the Animal Management and Ethics Committee of Jilin Agricultural University and followed the National Guiding Principles for the Welfare of Laboratory Animals strictly. If the animal developed dyspnoea, haemorrhagic diarrhoea, or showed signs of mortality, they were euthanized immediately by CO_2_ inhalation.

### Bacterial strains

*STM* was provided by Jilin Agricultural University. For the *STM* used to infect mice, it was first cultured overnight in LB medium, then passaged in fresh LB medium containing 0.3 M sodium chloride until the OD_600_ value reached, followed by two washes with PBS buffer. The bacterial sediment was resuspended in PBS, and the final concentration of the *STM* suspension was adjusted to 1 × 10^7^ CFU/mL and stored for later use.

*LGG* (ATCC 53,103) was grown in De Man, Rogosa, and Sharpe (MRS) broth for 12 h at 37°C. After culturing overnight, the bacteria were inoculated 1:100 in fresh MRS broth and grown under anaerobic conditions until reaching the mid-log phase. Then, the colonies were counted, and the cell density was adjusted to 1 × 10^8^ CFU/mL.

### *STM* Infection Experiment and Sample Collection

Six-week-old mice were randomly divided into two groups, each consisting of 10 mice. The PBS group was fed 200 μL of PBS for 8 consecutive days. The *STM* group was fed 100 μL (1 × 10^7^ CFU/mL) of *STM* suspension daily for 8 consecutive days. Then, mouse weight changes and survival rates were recorded. Following this, an *STM* infection experiment was repeated with another 20 six-week-old mice, and the mice were euthanized four days later. The small intestine and colon were collected for length measurements and formalin fixation.

### Pathological sections and indirect immunofluorescence

Histopathological analysis was carried out on small intestine, colon, and spleen samples collected after infection. All samples were fixed with 4% paraformaldehyde, and sections were stained with haematoxylin and eosin to examine pathological changes. For immunofluorescence, diluted primary antibodies VILLIN (Abcam, ab130751), EpCAM (Abcam, ab213500), LGR5 (Abcam, ab75850) were added and incubated overnight at 4°C in the dark, followed by washing. Secondary antibodies AF594 anti-rabbit (Abcam, ab150080) was incubated for 1 hour at 4°C in the dark. After washing, nuclear staining was performed using PBS diluted DAPI at 1:5000 at room temperature in the dark for 10 minutes, allowing for nuclear staining. Following another wash, slides were mounted for microscope examination.

### Cell separation

Cell samples obtained from the the mouse intestine were subjected to subsequent flow assay and in vitro cell culture and qPCR experiment. Firstly, after euthanasia of mice, the small intestine and Colon were dissected longitudinally, rinsed with PBS and divided into 1 cm sized intestinal fragments, which were then transferred to the separation solution (15 mL of RPMI-1640, 1% penicillin and streptomycin (Sigma, V900929), 1% HEPES (Sigma, H3375), 2.5 mM EDTA (Sigma, E8008), 1 mM DTT (Sigma, 3 December 3483), and 1% heat-inactivated FBS (Sigma, F8318)) and incubated for 28 minutes in a shaking incubator at 37°C and 200 rpm, and then removed. After incubation for 18 minutes in a shaking incubator at 37°C and 180 rpm, the intestinal fragments were obtained rinsed and continued into the enzyme digestion solution (8 mL RPMI-1640 medium, 1% penicillin and streptomycin, 1% HEPES, 20 mg collagenase IV (Sigma, V900893-1 G), 0.5 mg DNase I (Sigma 10,104,159,001), and 1% FBS), and incubated for 25 minutes in a shaking incubator at 37°C and 220 rpm before being removed, and then filtered through a 70-μm cell strainer to get the LPL cells in the mouse intestine. Finally, percoll (GE Healthcare 17,089,101) was used for density gradient centrifugation to obtain lymphocytes for subsequent experiments.

### Flow cytometry and antibody information

First, antibodies were added to tubes containing 1 × 10^6^ cells, mixed thoroughly and stained for 30 min at 4°C under dark conditions. Then, add 1 mL of PBS, centrifuge at 2000 r pm and 4°C for 5 min and discard the supernatant. The cells can then optionally be fixed and permeabilized, and after permeabilization the antibody can be used to continue the staining. The staining is completed and detected using a flow cytometric analyser (BD).

BD Pharmingen: Fixable Viability Stain 780 (L/D) (565388), purified rat anti-mouse CD16/CD32 (Mouse BD Fc Block) (553142), γδ T (Biotin) (553176), CD19 (Biotin) (553784), CD11b (Biotin) (557395), TCRβ (Biotin) (553168), Ly6G/C (Biotin) (553124), TER-119 (Biotin) (553672), streptavidin protein (APC-cy7) (554063), CD45 (FITC) (551874), CD127 (PE-cy7) (560733), RORγt (PE) (562607), and GATA3 (BV421) (563349), IL-22 (Alexa Fluor 647) (567160), MHCII (PE) (558593), CD11c (FITC) (553801), IL-23 (Alexa Fluor 647) (565317).

### *LGG* inhibited the *STM* infection

We divided the 5 Weeks old mice into three groups of 10 animals each. The PBS group were fed PBS for 14 days as the control group. Then the PBS+*STM* group was first fed PBS for 7 days, and then received *STM* infection. The *LGG*+*STM* group continued to be fed with *LGG* for 7 days, each mouse is fed 100 μL (1 × 10^7^CFU) *LGG* per day, and then received *STM* infection. Next, mouse weight changes and survival rates were recorded for 8 days. The PBS+*STM* group and *LGG*+*STM* group experiments were repeated, and the mice were euthanized on the fourth day of infection with *STM*, and then intestinal tissues and cells were collected for subsequent experiments.

### ELISA and qPCR experiments

Cytokine protein and total RNA were extracted from the mouse intestine and secretion of IL-22 was detected using the ELISA kit (MEIMIAN, MM-0892 M2). Next, total RNA was extracted, and 1 mg of RNA was reversed into cDNA by reverse transcriptase (Promega) which reverse transcribed Moloney mouse leukaemia virus (M-MLV). In the real-time qPCR system of Biological System 7500, qPCR was performed using SYBR green mixture (Takara). The average mRNA fold changes were calculated by 2-ΔΔCT method and compared with the control group.

Primer design: IL-22 (NM_016971.2),

FORWARD: CCTGCTTCTCATTGCCCTGTGG,

REVERSE: AAGGTGCGGTTGACGATGTATGG.

IL-23 (NM_031252.2),

FORWARD: AGCCAACTCCTCCAGCCAGAG,

REVERSE: CGCTGCCACTGCTGACTAGAAC.

### 16S rRNA-seq experiment

We performed a 16S rRNA sequencing of the intestinal contents of the mice. Novogene Co., for providing technical services such as detecting and analysing of 16S rRNA-seq raw data.

### Flow sorting of ILC3 and DC

Single-cell suspensions were incubated with antibodies including Lin (γδ T, CD19, CD11b, TCR-β, Ly6G/C, TER-119), L/D, MHC-II, CD11c, CD45, etc. DCs were sorted as L/D^−^CD11c^+^MHCII^+^ cells, and ILC3 were obtained through Lin^−^L/D^−^CD45^+^ sorting, noting that Lin^−^L/D^−^CD45^++^ indicates ILC2.

### In vitro stimulation culture of primary cells

ILC3s were seeded in a 24-well plate at a density of 5 × 10^6^ cells per well. HK-*LGG* (50 μL), *LGG* supernatant (50 μL), and DCs (5 × 10^5^) were added to the culture medium with ILC3 and incubated for 8 hours before being analysed by flow cytometry and qPCR.

### Organoid extraction and culture

1. After euthanizing the mouse, the small intestine was removed, mesentery and fat were discarded, and the intestinal segment was longitudinally opened and washed with cold PBS until the supernatant was clear. The intestinal segments were cut into 2 mm pieces and gently washed with cold PBS, then added to 15 mL of crypt isolation solution (1 mM EDTA in PBS). Incubated at room temperature for 30 minutes.
2. The crypt isolation solution was discarded, and 10 mL of DPBS was added to repeatedly pipette the fragments. After the fragments settled, the supernatant was collected through a 70 μm cell strainer into a 50 mL centrifuge tube, labelled as 1, and this step was repeated four times. The 3rd and 4th filtrates were centrifuged at 300×g for 5 minutes, and the supernatant was discarded. The pellet was resuspended in 1 mL of DME/F12 + 1% P/S and transferred to a 1.5 mL centrifuge tube, then centrifuged at 200×g for 3 minutes, and the supernatant was discarded.
3. The pellet was mixed with 250 μL of complete medium and 250 μL of Matrigel (operation on ice), mixed well by pipetting. 50 μL was pipetted into the centre of a well in a 24-well plate and incubated in a culture incubator for 30 minutes. Then, 500 μL of complete culture medium (STEMCELL #6000) was added to each well, and 500 μL of PBS was added to the remaining wells.
4. When organoids begin to bud, they should be passaged. First, the old culture medium is removed, and 2 mL of DME/F12 is added for pipetting up and down before collection into a centrifuge tube. After centrifugation, the supernatant is discarded, and the pellet is resuspended in complete culture medium and Matrigel for further cultivation.

### Co-culture Model of ILC3 and intestinal organoids

HK-*LGG*, DCs, and ILC3 are added to the organoid culture medium to observe their effects on the growth and development of organoids. Medium 1 is the organoid culture medium. Medium 2 consists of RPMI-1640 (1% penicillin and streptomycin, 1% HEPES, 10% FBS). HK-*LGG*, DCs, and ILC3 can be added to Medium 2.

### Statistical analysis of data

Flow cytometry results were analysed using FlowJo version 10.8.1. Graphs were plotted using GraphPad Prism version 8.0.2 software. Data analysis was carried out using one-way ANOVA to compare differences between control and experimental groups. (*p* < 0.05 is denoted by *; *p* < 0.01 by **; *p* < 0.001 by ***).

## Results

### *STM* Infection Causes Mortality and Intestinal Lesions in Mice

**Figure 1.**
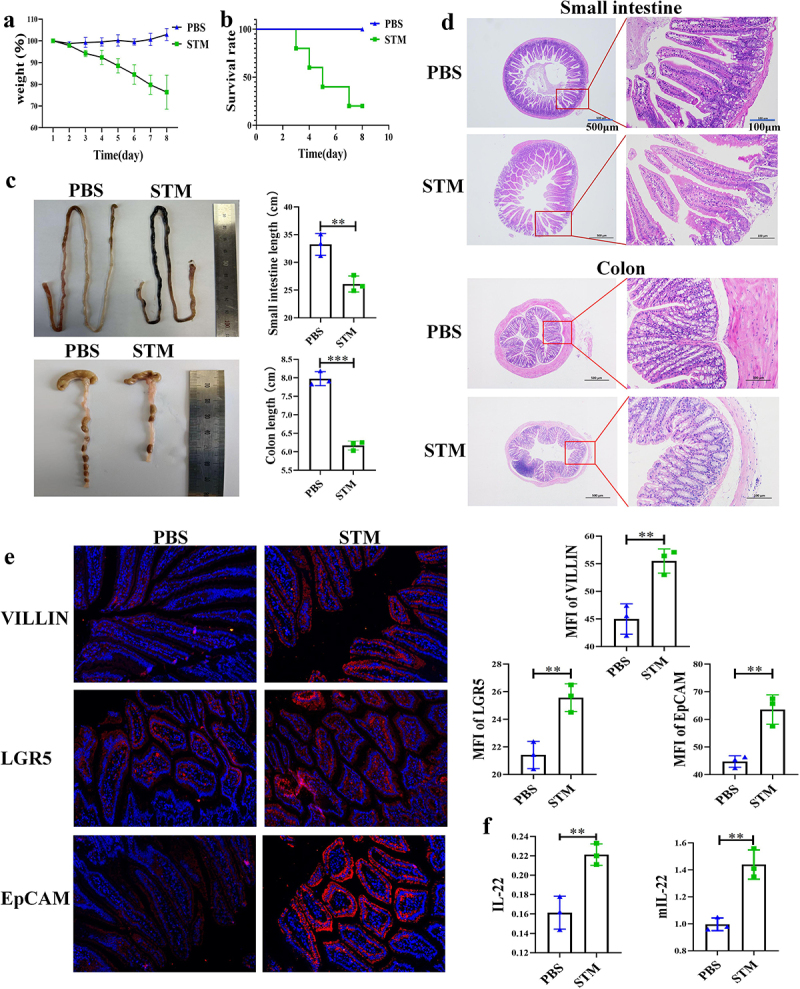
Fig. 1 | STM infection caused death and intestinal lesions in the mice a: STM infection leads to decreased host weight in mice (*n* = 5). b: STM infection results in mortality of mice (*n* = 5). c: STM infection causes the shrinking of the small intestine and colon in mice. d: STM infection induces pathological changes in the small intestine and colon of mice. e: after STM infection, mice exhibit elevated expression of VILLIN, LGR5, and EpCAM proteins in the small intestine. f: following STM infection, there is a significant increase in the secretion of IL-22 protein and transcription of mIL-22 in the intestinal tract of mice.

This study found that mice infected with *STM* exhibited weight loss and even death. The body weight of mice in the PBS group increased, while that of mice in the *STM* group significantly decreased, with mortality observed on the third day and a survival rate of only 25% by the eighth day (Figure 1a,b). *STM* infection also led to the atrophy of the small intestine and colon in mice. The length of the small intestine in the PBS group was about 33 cm, and the colon was about 8 cm. In contrast, the small intestine in the *STM* group was about 26 cm, and the colon was about 6 cm (Figure 1c). In order to explore the immune changes in the mouse gut during this process, we repeated the infection experiment and euthanized the mice on the fourth day. Pathological sections revealed tissue damage in the small intestine and colon of mice in the *STM* group, including villi fracture, thinning of the intestinal wall, and extensive infiltration of red blood cells and lymphocytes (Figure 1d). Immunofluorescence experiments identified significant expression of intestinal villin protein, stem cell differentiation protein LGR5, and epithelial cell marker protein EpCAM in the intestines of *STM*-infected mice (Figure 1e). ELISA and qPCR analyses showed that the secretion of IL-22 and the transcription level of the mIL-22 gene in the intestines of mice in the *STM* group were significantly higher than in the PBS group (Figure 1f). The experimental results indicate that *STM* infection in the mouse intestine causes severe intestinal damage and endangers the lives of the mice, with higher levels of IL-22 being secreted in the intestine.

### STM Infection Leads to Significant Changes in Mouse gut microbiota and IL-22 Secretion by ILC3

**Figure 2.**
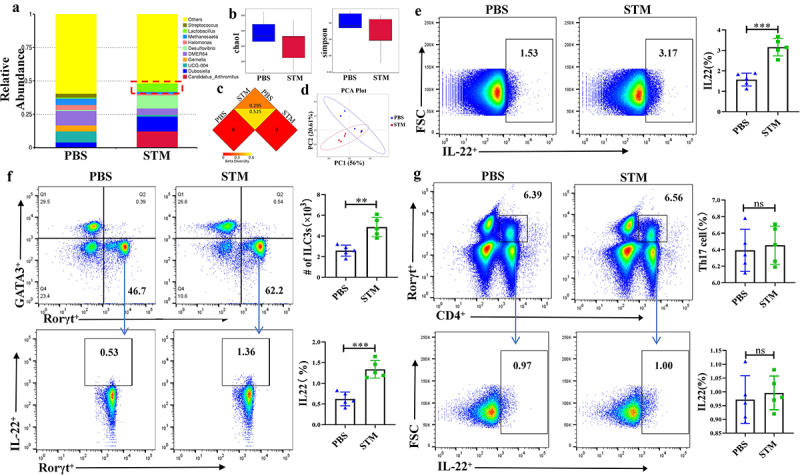
*STM* infection leads to significant changes in the gut microbiota and IL-22 secretion by ILC3 in mice.a: Changes in *Lactobacillus* Genus in the intestinal tract of piglets (*n*=5). b: Box plot of alpha diversity analysis (Chao1, Simpson index). c: Heatmap of beta diversity analysis. d: Inter-group PCA analysis. e: Differences in IL-22 secretion by CD45^+^ immune cells between the PBS and *STM* groups. f: Flow cytometry analysis of the number of ILC3 cells and the level of IL-22 secretion in the lamina propria of the mouse small intestine. g: Flow cytometry analysis of the number of TH17 cells and the level of IL-22 secretion in the lamina propria of the mouse small intestine.

The results of phylum level analysis from 16s-RNA sequencing found that infection with *STM* resulted in increased proportion of *Firmicutes*, *Proteobacteria* and *Actinobacteriota*. The genus level analysis found that the proportion of *Lacticaseibacillus* in the gut increased significantly (Figure 2a). Box plots of α-diversity analysis indicated that the abundance and diversity of intestinal microbiota significantly decreased after *STM* infection (Figure 2b). β-diversity analysis showed significant differences in species diversity between the PBS and *STM* groups (Figure 2c). PCA analysis Also shows a difference in the PBS and *STM* groups (Figure 2d).

Flow cytometry detection revealed that IL-22 expression mainly originated from CD45^+^ immune cells (Figure 2e), with gating strategy shown in Fig. s1A. Lymphocytes known to secrete IL-22 primarily include ILC3 and CD4^+^ T cells. To determine the source of IL-22, we separately measured the levels of IL-22 secreted by ILC3 and CD4^+^ T cells. Results showed a significant increase in both the number of ILC3 cells and the IL-22 they secreted (Figure 2f), with gating strategy shown in Fig. s1B. In every million lymphocytes, the absolute number of ILC3 in the PBS group was 47,800, compared to 25,600 in the *STM* group. Meanwhile, the secretion of IL-22 by CD4^+^ T cells showed almost no change (Figure 2g), with gating strategy shown in Fig. s1C. The significant increase of *Lacticaseibacillus* in the early stage of STM infection, as well as the secretion of IL-22 by ILC3, led us to speculate that *Lacticaseibacillus* may play a key role in inhibiting STM infection

### Feeding mice with *LGG* can inhibit STM infection

**Figure 3.**
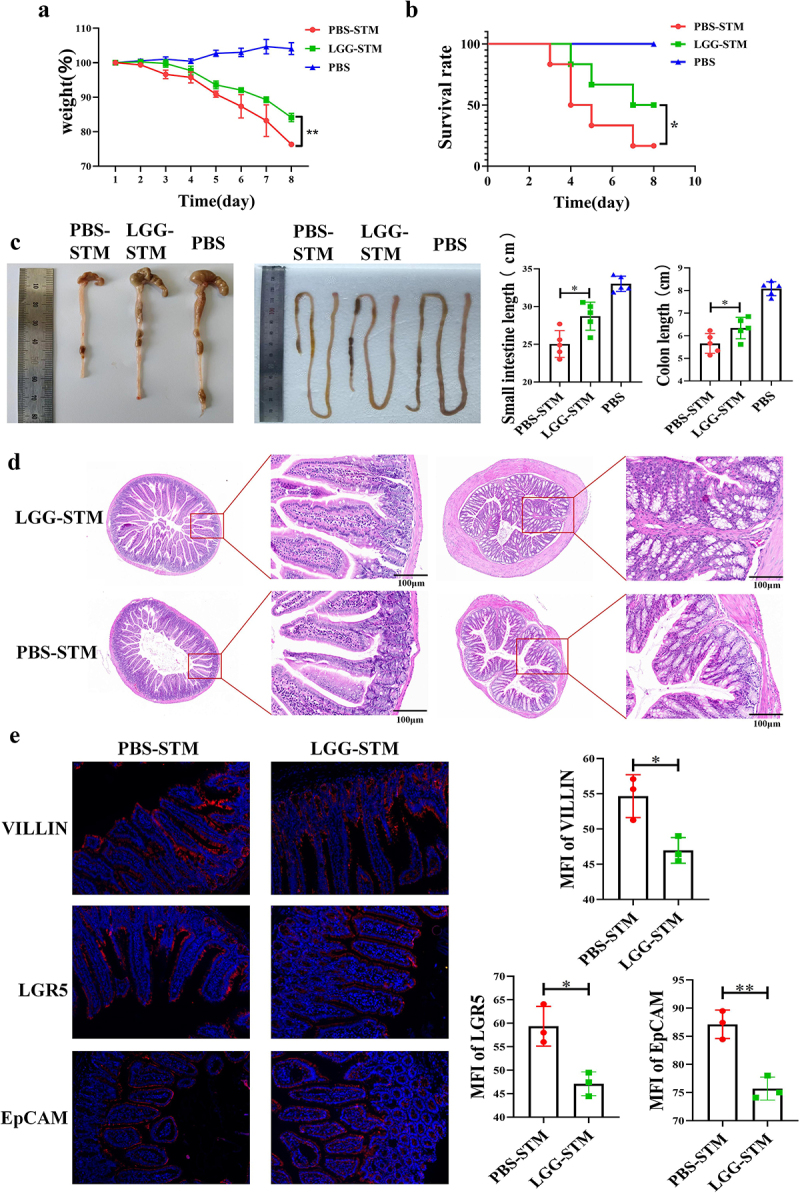
LGG can help mice resist STM infection.a: Feeding *LGG* significantly alleviates weight loss in mice after *STM* infection. b: Feeding *LGG* significantly reduces mortality in mice after *STM* infection. c: Feeding *LGG* significantly alleviates the shrinking of the small intestine and colon in mice after *STM* infection. d: Feeding *LGG* can significantly alleviate the pathological changes in the small intestine and colon in mice after *STM*infection. e: Feeding *LGG* significantly reduces the expression of VILLIN, LGR5, and EpCAM proteins in the small intestine of mice after *STM* infection.

To explore the specific role of *Lacticaseibacillus* in the intestinal *STM* infection, we orally administered *LGG* to mice and then infected them with *STM* after 7 days. By analysing the mortality and body weight changes of the mice, it was found that compared to the *LGG-STM* group, mice in the PBS-*STM* group experienced more severe weight loss (Figure 3a) and had a lower survival rate (Figure 3b). The lengths of the small intestine and colon in the *LGG-STM* group were also found to be closer to those of the PBS group (Figure 3c). Analysis of the *STM* load in faeces revealed a significantly lower number of *STM* in the faeces of mice in the *LGG-STM* group compared to the PBS-*STM* group (Fig. s2A). The degree of pathological changes in the intestines of mice in the *LGG-STM* group was also significantly lower than that in the PBS-*STM* group, with the villi in the jejunum of the *LGG*+*STM* group mice showing shortening and atrophy, and epithelial cells showing mild lesions. The villi in the jejunum of PBS+*STM* group mice exhibited shortening, fragmentation, and breaking, with vacuolization, necrosis, and shedding of the intestinal epithelial cells, among other histopathological changes (Figure 3d). Immunofluorescence experiments further revealed that the expression levels of VILLIN and LGR5 proteins in the small intestine of mice in the *LGG*+*STM* group were lower than in the PBS-*STM* group (Figure 3e). These results suggest that feeding *LGG* can significantly reduce the mortality and intestinal lesions caused by *STM* infection in mice.

### Feeding *LGG* Promotes the Development of ILC3 and Secretion of IL-22 in the Mouse Intestine

**Figure 4.**
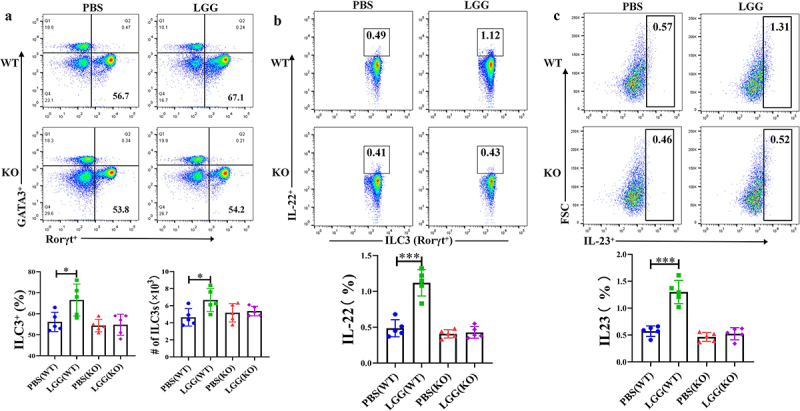
LGG promotes activation of ILC3 cells and is associated with IL-23 expression by TLR2 and DCs.a: Flow cytometry analysis of changes in ILC3 in the lamina propria of WT and KO mice after *LGG* feeding. b: Flow cytometry analysis of the level of IL-22 secretion by ILC3 in the lamina propria of WT and KO mice after *LGG* feeding. c: Flow cytometry analysis of the level of IL-23 expression by DCs in the lamina propria of WT and KO mice after *LGG* feeding.

We further investigated the effect of feeding *LGG* on the activation of intestinal ILC3 in mice and used TLR2^−/−^ mice to verify the importance of TLR2 in this process. Flow cytometry results showed that feeding *LGG* increased the number of ILC3 in the mouse intestine and promoted the secretion of IL-22. However, after feeding *LGG* to TLR2^−/−^ mice, the activation effect of *LGG* on ILC3 was absent (Figure 4a,b), indicating a key role of TLR2 in the activation of ILC3 by *LGG*. It is known that TLRs are mainly expressed on the surface of DCs in the mouse intestine. We also conducted flow cytometry analysis on DCs (gating strategy shown in Fig. s2B), and results showed significant differences in the expression of IL-23 by DCs in the lamina propria of TLR2^−/−^ mice and wild-type mice after feeding *LGG* (Figure 4c). In summary, feeding *LGG* to wild-type mice significantly promoted the secretion of IL-23 by DCs in the lamina propria, and concurrently, the number of ILC3 and the secretion of IL-22 were also significantly increased, while feeding *LGG* to TLR2^−/−^ mice did not induce these changes. These results indicate that *LGG* may interact with DCs and promote the secretion of IL-23. Next, we conducted in vitro experiments to verify whether IL-23 secreted by DCs stimulated by *LGG* could promote the activation of ILC3 and the secretion of IL-22.

***HK-LGG promotes IL-22 secretion by ILC3 through DCs***

**Figure 5.**
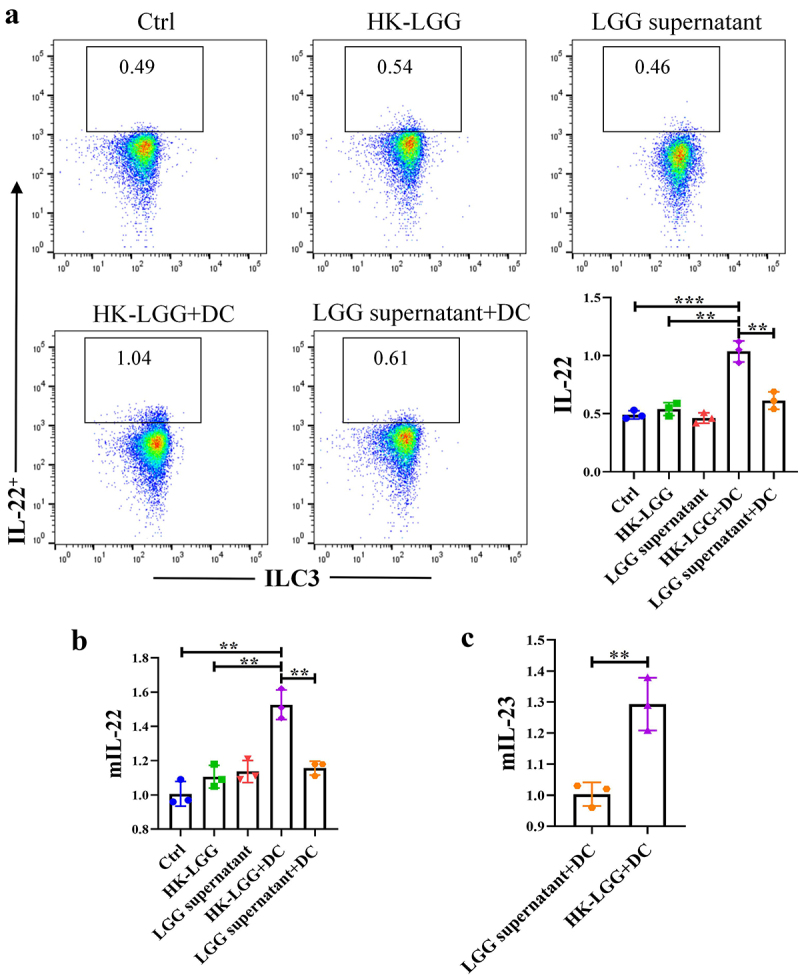
*HK-LGG* activates ILC3 by promoting IL-23 secretion from DCs.a: Flow cytometry analysis of the activating effect of HK-*LGG*, *LGG* supernatant, and DCs on IL-22 secretion by mouse ILC3. b: Transcription levels of the IL-22 gene in ILC3 under stimulation by different groups. c: Differential levels of IL-23 secretion promoted by HK-*LGG* and *LGG* supernatant from DCs.

Initially, we obtained DCs and ILC3, and the flow cytometry sorting strategy is shown in Fig. s3A/B. Subsequently, an in vitro co-culture model was constructed (Fig. s3C), followed by control experiments using *LGG* culture supernatant and HK-*LGG*. In vitro studies found that without DCs, neither HK-*LGG* nor *LGG* supernatant could promote IL-22 secretion by ILC3. However, when DCs were added, the HK-*LGG* could promote IL-22 secretion by ILC3 (Figure 5a). Next, we measured the transcription levels of mIL-22 in co-cultured ILC3 cells and mIL-23 in DCs via qPCR experiments. It was found that the transcription of mIL-22 in the culture medium of the HK-*LGG*+DC group was significantly higher than in other groups (Figure 5b), while the transcription level of mIL-23 in the *LGG* supernatant+DC group was significantly lower than in the HK-*LGG*+DC group (Figure 5c). These results indicate that HK-*LGG* can promote the secretion of IL-23 by DCs, and the IL-23 secreted by DCs can further promote the secretion of IL-22 by ILC3. To explore whether IL-22 could further enhance the function of the intestinal mucosal immune barrier in this process, we conducted further experimental studies using intestinal organoids.

### IL-22 Regulates the Immune Barrier Function of Intestinal Epithelium

**Figure 6.**
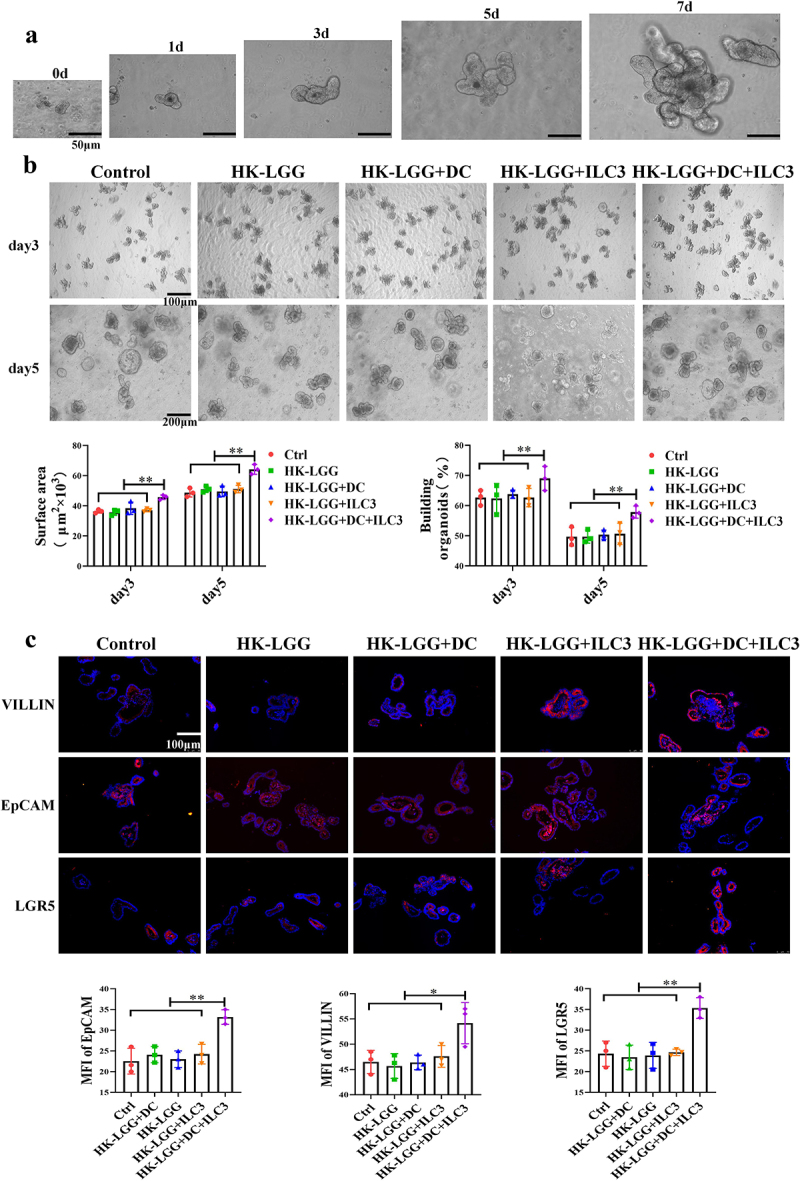
*HK-LGG* can promote the development of mice intestinal organoids through activating ILC3s to secrete IL-22.a: Crypts isolated from mice intestines were cultured in vitro, and the images depict the changes in organoid size over an 7-day period. b: Effects of HK-*LGG*; HK-*LGG+*DC; HK-*LGG+*ILC3 and HK-*LGG+*DC+ILC3 on the growth of intestinal organoids. Measure the size (surface area) and budding rate (building organoids) of the organs on the third and fifth days respectively. c: Immunofluorescence experiment demonstrating the expression of proteins such as Villin, EpCAM, LGR5 between different groups.

Firstly, we successfully established an in vitro culture model of mouse intestinal organoids (Fig. s3D), with isolated intestinal crypts approximately 10 μm in size, cultured in a matrix gel. Budding began in large numbers on day 3, and by day 7, they had grown into mature entities approximately 100 μm in diameter (Figure 6a). Next, we co-cultured HK-*LGG*, DCs, ILC3, and organoids. The results showed no significant developmental changes in the organoids in the Ctrl group, HK-*LGG* group, HK-*LGG*+DC group, and HK-*LGG*+ILC3 group; however, the intestinal organoids in the HK-*LGG*+DC+ILC3 group growed faster. On days 3 and 5, we assessed the volume and budding of the organoids, finding that budding and growth in the HK-*LGG*+DC+ILC3 group were significantly higher than in the other groups (Figure 6b). Immunofluorescence experiments revealed that the expression of villin, epithelial protein, and LGR5 protein in the organoids of the HK-*LGG*+DC+ILC3 group was also higher than in the ILC3 group and the DC+ILC group (Figure 6c). These results demonstrate that HK-*LGG* can promote DCs to secrete IL-23, which then encourages ILC3 to secrete IL-22, and IL-22 ultimately promotes the development of intestinal organoids.

**Figure 7.**
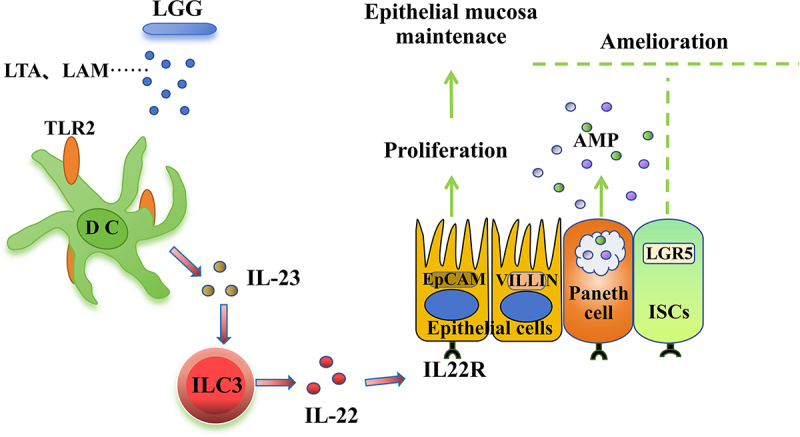
The pathway of HK-*LGG* enhances the intestinal mucosal immune barrier in mice.

Studies have reported that components of the *LGG* cell wall can be recognized by TLR2, stimulating DCs to secrete IL-23^7^. Based on existing research reports and our experimental results, a regulatory pathway diagram was created: LTA and LAM from HK-*LGG* can be recognized by TLR2-expressing DCs leading to the secretion of IL-23, which acts on ILC3 to promote the secretion of IL-22. IL-22 can perform multiple functions, including promoting the development of organoids and activating epithelial cells and Paneth cells (Figure 7).

## Discussion

*STM* is a facultative intracellular pathogen that can actively invade and replicate in host cells [28]. *STM* has a wide range of hosts, including humans, various livestock, wild animals, etc., and is mainly transmitted through contaminated food or water sources [29,30]. Studies have found that intestinal mucosal immunity can trigger local inflammation when inhibiting *STM* infection [31]. Recent studies have shown that the host immune system will produce specific cytokines to rapidly inhibit *STM* infection, among which IL-22 is one of the most upregulated cytokines in the intestine [32]. This study found that mice infected with STM have increased abundance of Lacticaseibacillus in the intestine and intestinal lesions, while there was a significant increase in ILC3 and the secretion of IL-22 in the intestinal lamina propria. These results suggest that *Lacticaseibacillus* in the mouse intestine may play an important role during STM infection, potentially regulating ILC3 to secrete IL-22. Thus, the regulatory relationship between the microbiota and ILC3—whether it is a positive or negative feedback mechanism – warrants further exploration.

Intestinal microbiota play a crucial role in the development and maintenance of the host’s immune system, especially in regulating the development and differentiation of lymphocytes within the intestinal lamina propria [33,34]. In addition, the gut microbiota promotes the development of early B lymphocytes in the lamina propria of the mouse small intestine [33,34]. Preclinical studies and clinical practice have shown that the use of probiotics can limit the overgrowth of pathogenic bacteria and control the host’s pathological processes [35]. Among them, *LGG* is one of the most widely used probiotic strains and has long been recognized for its beneficial effects on human health [36,37]. To explore the role of *Lacticaseibacillus* in *STM* infection, we orally administered the model probiotic *LGG* to mice before subjecting them to *STM* infection. The results showed that feeding *LGG* significantly alleviated the symptoms of *STM* infection in mice.

TLR2 recognizes and responds to threats early in bacterial infections and can influence the downstream immune response to the host’s benefit or detriment [38]. Research has reported that DCs expressing TLR2 play an important role in the regulation of intestinal microbiota [39], particularly lipoarabinomannan (LAM) and lipoteichoic acid (LTA) from *LGG*, which can activate DCs to secrete IL-23 through TLR2 [40,41]. Subsequently, ILC3 can produce the cytokine interleukin IL-22 in response to IL-23 signalling [42]. To further explore the activating effect of *LGG* on the intestinal immune barrier, we constructed an in vitro cell co-culture model for validation. Our results found that HK-*LGG* can activate DCs via TLR2 and promote the secretion of IL-23, which in turn can enhance the proliferation of ILC3 cells and the secretion of IL-22.

Some studies have reported that the intestinal microbiota is important for IL-22 production in the intestine, but the underlying regulatory mechanism remains unclear [43]. In the mouse intestine, research has documented the crucial role of the IL-22-IL-22 R signalling axis in immune responses and mucosal surface barrier functions [44]. Studies also report that IL-22 can promote epithelial cell activation and the expression of antimicrobial peptides through the activation of the STAT3 signalling pathway [45]. Multiple studies have underscored the importance of IL-22 produced by ILC3 in maintaining intestinal homoeostasis [46]. We constructed a mouse intestinal organoid model to further validate the impact of IL-22 on organoid development, showing that IL-22 can also act on Paneth cells, stem cells, and epithelial cells in organoids to promote their growth and development.

*Salmonella* infection has caused serious health problems for people in developing countries. Studies have found that the use of yeast probiotics protects against Salmonella infections [47]. Studies have also found the role of Sphingolipids on Innate Immunity to Intestinal *Salmonella* Infection [48]. In this study, we discovered that LAM and LTA from *LGG* can activate DCs and secrete IL-23 through TLR2, and IL-23 can further activate ILC3 to secrete IL-22, maintaining intestinal immune homoeostasis. While this work provides a theoretical basis and experimental foundation for the development of intestinal health regulatory products and treatment strategies, the complexity of the microbial species in the intestine leaves unanswered whether LAM and LTA from other microbial sources can also exert similar immunomodulatory effects.

In recent years, research into the interactions between microbiota and the immune system has garnered considerable attention. On one hand, the immune system can regulate and shape the microbial flora [49]. On the other hand, the colonized microbial flora can promote the development of the host’s immune system and provide signals for subsequent immune responses [50]. However, to date, our understanding of the interactions between microbiota and the immune system remains significantly limited, and unravelling these mysteries requires coordinated innovation across multiple disciplines. Our work is just the beginning, and in the future, we will delve deeper into exploring the mechanisms of interaction between *LGG* and intestinal immune cells.

## Supplementary Material

Fig._s1.jpg

Fig._s3.jpg

Fig._s2.jpg

## Data Availability

The data that support the findings of this study are openly available in https://figshare.com/s/641045c425176601b3b6, DOI: 10.6084/m9.figshare.25826965. And 16S rRNA-seq data in https://www.ncbi.nlm.nih.gov/bioproject, reference number is [PRJNA1073044].
