# Supplementary figures and images for: LGG promotes activation of intestinal ILC3 through TLR2 receptor and inhibits salmonella typhimurium infection in mice
Source: Virulence. 2024 Jul 30;15(1):2384553. doi: 10.1080/21505594.2024.2384553 (PMC11296546; doi:10.1080/21505594.2024.2384553)

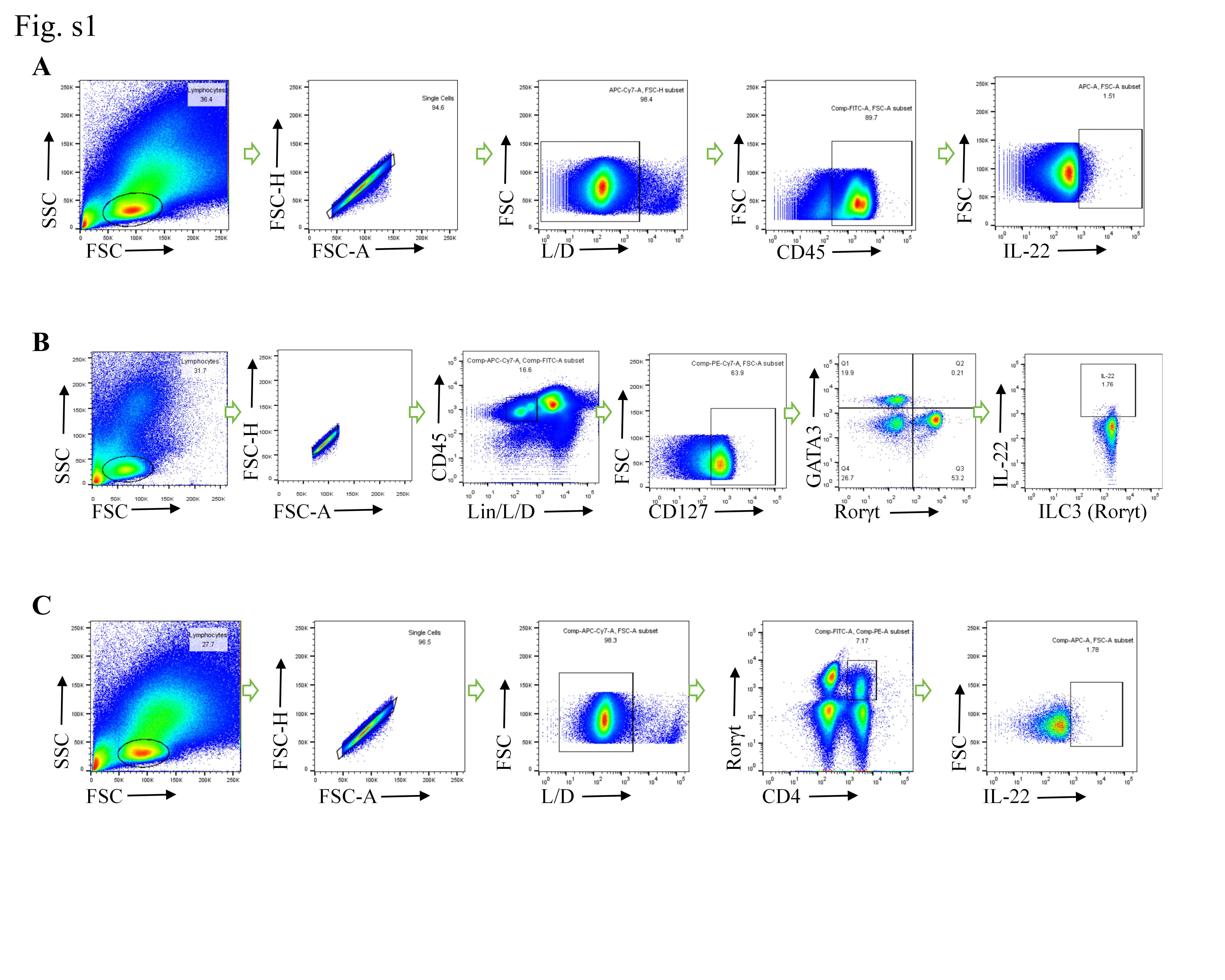

Supplement: Fig._s1.jpg [file KVIR_A_2384553_SM6869.jpg]

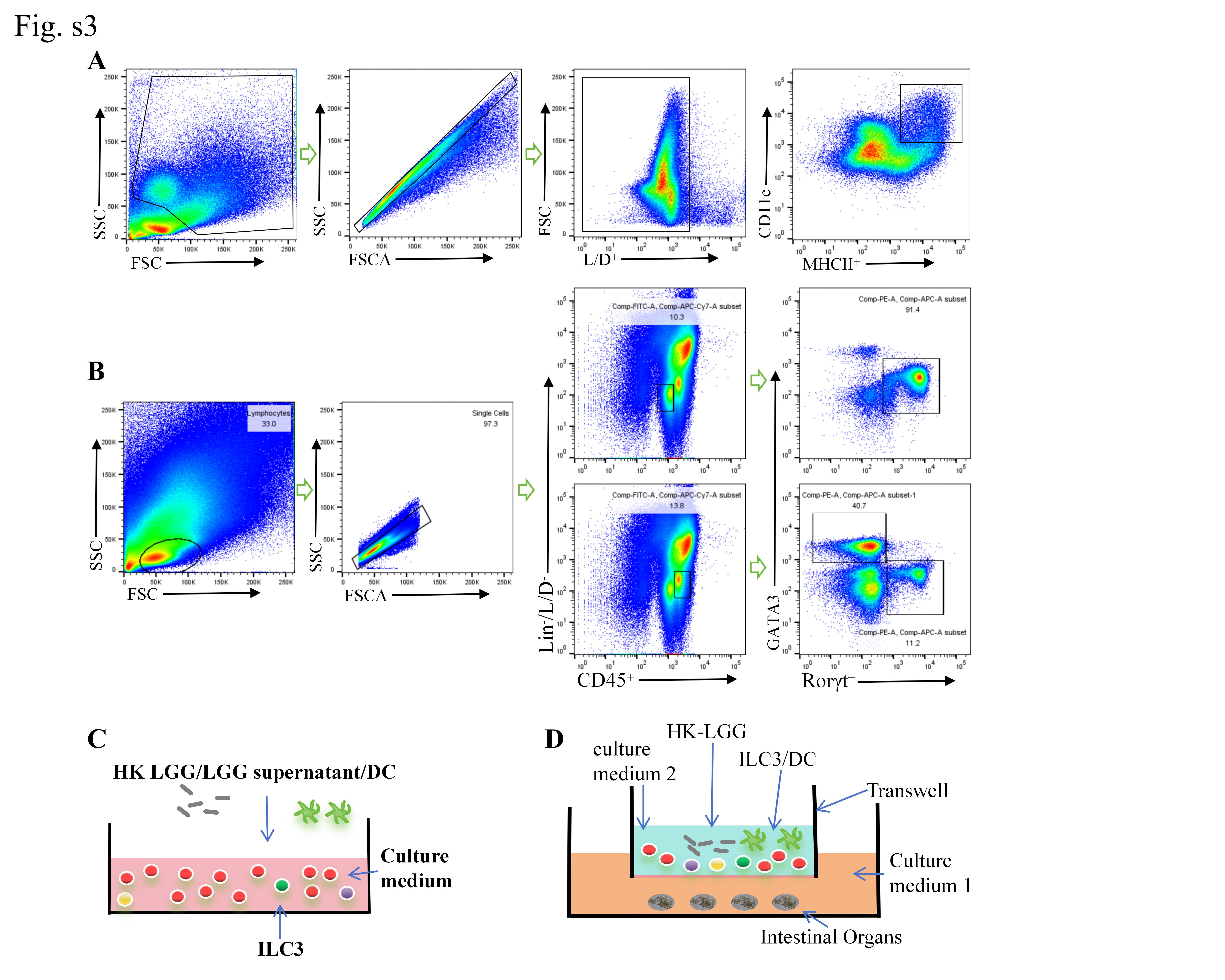

Supplement: Fig._s3.jpg [file KVIR_A_2384553_SM6868.jpg]

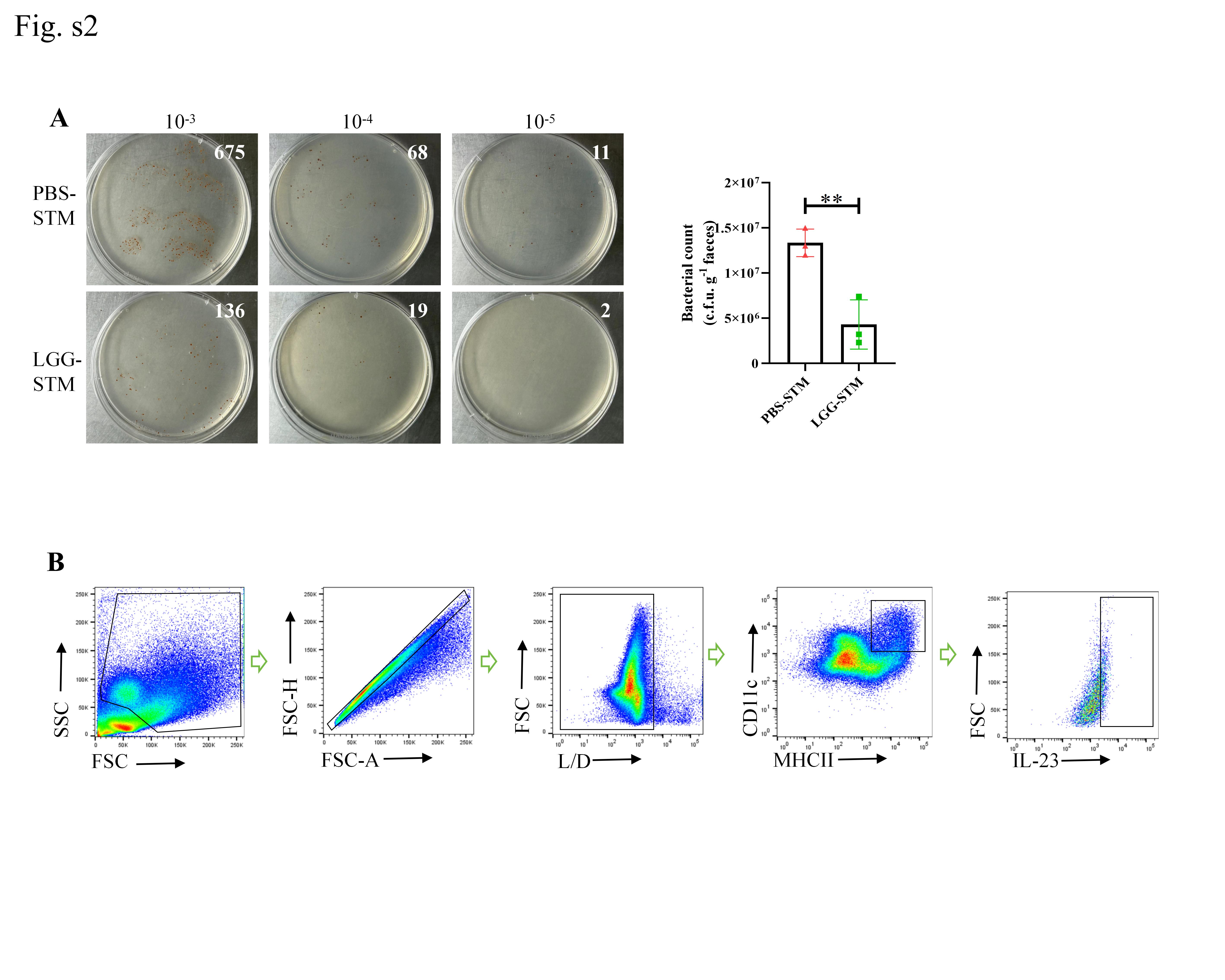

Supplement: Fig._s2.jpg [file KVIR_A_2384553_SM6867.jpg]
